# Analysis of Superspreading Potential from Transmission Clusters of COVID-19 in South Korea

**DOI:** 10.3390/ijerph182412893

**Published:** 2021-12-07

**Authors:** Hyojung Lee, Changyong Han, Jooyi Jung, Sunmi Lee

**Affiliations:** 1 Department of Statistics, Kyungpook National University, Daegu 41566, Korea; hjlee@knu.ac.kr; 2 Department of Applied Mathematics, Kyung Hee University, Yongin 17104, Korea; cyhan@khu.ac.kr; 3 Department of Biostatistics, Korea University, Seoul 02841, Korea; wjdwndl8113@naver.com

**Keywords:** SARS-CoV-2, COVID-19, statistical model, superspreading events, cluster-induced transmissions, cluster-specific reproduction number

## Abstract

The COVID-19 pandemic has been spreading worldwide with more than 246 million confirmed cases and 5 million deaths across more than 200 countries as of October 2021. There have been multiple disease clusters, and transmission in South Korea continues. We aim to analyze COVID-19 clusters in Seoul from 4 March to 4 December 2020. A branching process model is employed to investigate the strength and heterogeneity of cluster-induced transmissions. We estimate the cluster-specific effective reproduction number $R_{\mathrm{eff}}$ and the dispersion parameter κ using a maximum likelihood method. We also compute $R_{m}$ as the mean secondary daily cases during the infection period with a cluster size *m*. As a result, a total of 61 clusters with 3088 cases are elucidated. The clusters are categorized into six groups, including religious groups, convalescent homes, and hospitals. The values of $R_{\mathrm{eff}}$ and κ of all clusters are estimated to be 2.26 (95% CI: 2.02–2.53) and 0.20 (95% CI: 0.14–0.28), respectively. This indicates strong evidence for the occurrence of superspreading events in Seoul. The religious groups cluster has the largest value of $R_{\mathrm{eff}}$ among all clusters, followed by workplaces, schools, and convalescent home clusters. Our results allow us to infer the presence or absence of superspreading events and to understand the cluster-specific characteristics of COVID-19 outbreaks. Therefore, more effective suppression strategies can be implemented to halt the ongoing or future cluster transmissions caused by small and sporadic clusters as well as large superspreading events.

## 1. Introduction

A novel coronavirus disease (COVID-19) outbreak has been affecting a number of countries worldwide. Although a majority of countries have started their vaccination drives, the ongoing COVID-19 pandemic has reached 246 million infections, resulting in 5 million deaths, as of 31 October 2021 [1]. Due to complex factors including the relaxation of social distancing measures and new variants, South Korea has experienced four major waves of the pandemic. 

One of the common features in these four major waves is superspreading events or cluster-driven transmissions. In South Korea, the first confirmed case of COVID-19 was a 35-year-old female from China who reported on 20 January 2020, and the first wave occurred within a few months due to superspreading events (SSEs) related to a church cluster in the southeastern Daegu-Gyeongbuk (DG) area [2]. Since then, cluster-driven transmissions have continued to occur in the Seoul metropolitan region, such as at a customer service call center and nightclubs. Due to the significant breakouts in churches and a demonstration on 15 August, a massive outbreak in the Seoul metropolitan region occurred in mid-August, and the number of new cases rapidly increased. There have been additional three waves throughout the country. The fourth wave has expanded substantially as of October 2021, with 364,700 confirmed cases and 2849 deaths in South Korea [3,4,5].

Like other infectious diseases in the coronavirus family, such as SARS-CoV-1 and MERS-CoV, the current SARS-CoV-2 can spread rapidly in confined and crowded places with effective close contacts. Instead of a nationwide lockdown, the Korean government has enforced a series of social distancing measures [5,6] such as online worship and a ban on large social gatherings [3]. From 1 January to 4 December 2020, the total number of confirmed cases was 36,332, of which 9716 cases occurred in Seoul (26.74%). Major sources of infection include 4652 imported cases and 20,501 cluster-related infections, which accounted for more than 50% of all confirmed cases. Among all 20,501 people infected in the clusters in South Korea, 4928 of them were located in Seoul, accounting for approximately 24% of all cases. This is the second largest cluster after the DG area cluster. We focus on clusters in Seoul and investigate their characteristics, excluding imported cases using the publicly available data [5].

In mathematical epidemiology, the basic reproduction number $R_{0}$ is defined as the average number of secondary transmissions caused by a single primary case in a fully susceptible population. It is widely used to measure the severity of disease outbreaks. For COVID-19 confirmed cases in Japan and China, it has been estimated to be around 2–3 based on empirical data [7,8]. However, this $R_{0}$ alone is not sufficient to understand how cluster-driven transmissions can affect overall outbreaks. Therefore, it is necessary to provide additional information on the distribution of secondary cases or offspring to characterize SSEs and cluster-driven transmissions [9,10]. In general, the high overdispersion (i.e., high individual-level variation) in the offspring distribution, which can lead to SSEs, plays an important role in epidemic control. In other words, most cases do not contribute to the spread of the epidemic. The degree of overdispersion is quantified by the dispersion parameter κ, indicating the variance in the distribution of the number of secondary infections caused by a typical primary case. Lower values of κ indicate greater variance and tend to cause SSEs. Previous studies [8,11,12] have described the transmission dynamics of COVID-19 as highly overdispersed, reporting [11,13,14] the values of κ in the range of 0.1–0.6. Tariq et al. [15] analyzed the characteristics of the six largest clusters in Singapore showing that most secondary infections are caused by a small number of primary cases. 

In the present study, we aim to evaluate superspreading events (SSE) by investigating the characteristics of cluster-induced transmission dynamics of COVID-19 in Seoul, South Korea, from March to December 2020. We estimate the $R_{\mathrm{eff}}$ and κ values of all 61 clusters as $R_{\mathrm{eff}}$ = 2.26 (95% CI: 2.02–2.53) and κ = 0.20 (95% CI: 0.14–0.28), respectively. This shows strong evidence for the occurrence of superspreading events in Seoul. Our findings suggest that the reproduction number is larger than 1 in the clusters and the distribution of the number of secondary transmissions is highly overdispersed as κ<1. These findings are consistent with those of the earlier studies on SSEs observed in this pandemic. Moreover, the religious groups clusters had the largest value of $R_{\mathrm{eff}}$ among all clusters followed by workplaces, schools, and convalescent home clusters. Since most cases do not contribute to the expansion of transmission, effective reproduction numbers can be rapidly reduced by preventing SSEs. Compared to the previous SARS-CoV/MERS-CoV outbreaks, the current COVID-19 outbreak is found to have much higher $R_{\mathrm{eff}}$, but similar κ. The present study supports the possibility of highly overdispersed transmission of COVID-19 by analyzing the characteristics of the clusters. A highly overdispersed offspring distribution suggests that it is of a great importance to implement necessary measures to control SSEs. In light of the ongoing pandemic, therefore, the precise assessment of the transmission potentials of clusters should be continued to provide effective control measures of mass infections.

## 2. Materials and Methods

### 2.1. Epidemiological Data

We investigate the characteristics of cluster-induced transmission dynamics of COVID-19 in Seoul, South Korea. Owing to the hardworking of epidemiologists, the daily confirmed cases are publicly available from 4 March to 31 October 2021 [16] from Korea Disease Control and Prevention Agency (KDCA). In particular, we have focused on the identification of cluster-induced transmissions (a total of 9952) of COVID-19 in Seoul from 4 March to 4 December 2020. Our cluster analysis excludes imported cases who traveled from overseas. In general, a cluster is defined as a group of cases wherein each case can be epidemiologically associated with the others. In the present study, a cluster is defined as a group of at least 20 cases of COVID-19, including one or more primary cases. A previous study carried out cluster-induced transmission analysis with clusters with more than five cases for the first and the second waves of COVID-19 in the Seoul metropolitan area [17]. As the third wave of COVID-19 in South Korea began in November 2020 [3], our time window of interest spanned from the first wave to the early third wave. Therefore, we defined clusters as groups with more than 20 cases, otherwise the number of clusters becomes too large to analyze. As of 4 December 2020, we have identified 61 clusters in Seoul categorized into seven groups: religious group such as churches and temples, convalescent homes, hospitals, workplaces and schools, leisure facilities, nightclubs, and others.

### 2.2. Cluster-Specific Reproduction Numbers

We employed two types of cluster-specific basic reproduction numbers using the epidemiological data (confirmed cases of COVID-19). The first was the group’s effective reproduction number $R_{\mathrm{eff}}$ representing the mean number of secondary cases caused by an infected case. If $R_{\mathrm{eff}}<1$, transmission cannot take place, whereas there is potential for epidemic spread if $R_{\mathrm{eff}}>1$. Previous studies used a likelihood calculation based on branching process theory to infer the strength and heterogeneity of transmission [18]. The offspring distribution is the probability distribution for the number of secondary cases infected directly by each infectious individual. The negative binomial describes the offspring distribution, including the superspreading with a high degree of overdispersion. The number of transmissions caused by each new infection was assumed to follow a negative binomial distribution. The distribution was modeled with a mean of $R_{\mathrm{eff}}$ and dispersion parameter κ. The model of chain size distribution incorporates both $R_{\mathrm{eff}}$ and dispersion parameter *κ*, which indicates a measure of variability in the cluster size [15,19,20]. In other words, a higher degree of heterogeneity indicates that some individuals tend to spread the infection to a larger number of people (i.e., superspreaders). It means that a Poisson distribution was obtained as a special case of the negative binomial distribution as κ→∞ [12,21]. Therefore, the transmission was characterized by clusters of infection. We expected that $R_{\mathrm{eff}}$ would be lower and κ would be higher, which would indicate less transmission on average and lower risk of superspreading, respectively, if the control interventions were effective at preventing superspreading events. 

We modified the likelihood function described in previous studies [15,19]. The likelihood that an observed cluster of size j contains x primary cases is given by:

(1) $$\ell_{x\to j}^{C}\left(R_{\mathrm{eff}},\;\kappa ,x\right)=\frac{x}{j}\ell_{x\to \left(j-x\right)}\left(R_{\mathrm{eff}},\kappa \right),$$

where the likelihood of i infections causing j infections is given by:

(2) $$\ell_{i\to j}\left(R_{\mathrm{eff}},\kappa \right)=\frac{\Gamma \left(j+\kappa i\right)}{\Gamma \left(j+1\right)\Gamma \left(\kappa i\right)}{\left(\frac{\kappa }{R_{\mathrm{eff}}+\kappa }\right)}^{\kappa i}{\left(\frac{R_{\mathrm{eff}}}{R_{\mathrm{eff}}+\kappa }\right)}^{j},$$

where Γ is the gamma function. Although a primary case can be easily identified early in an outbreak, it is difficult to do so after a substantial number of cases has been confirmed. Therefore, we calculated the effective reproduction number and dispersion parameter by varying the number of primary cases; the number of primary cases varied between 5 and 20 cases because the number of primary cases is unknown. Lastly, we estimated $R_{\mathrm{eff}}$ and larger κ by using maximum likelihood estimation. 

Moreover, we computed the average number of secondary cases per day as the second type of the cluster-specific reproduction number. This was calculated using the average number of confirmed cases during the entire infectious period of a cluster *m*, denoted by $R_{m}$. This measurement may highlight the effectiveness of various interventions for cluster-induced transmission. In a similar manner, the previous studies analyzed the distribution of delay in days from symptom onset to confirmation by cluster size [14]. This was calculated using the cluster size j divided by the difference D between the reporting dates of the first case and the last case in the cluster:

$$R_{m}=\frac{j}{D}.$$

### 2.3. Ethical Considerations

Since the datasets used in our tables and figures are fully anonymized and do not include any personally identifiable information, no ethical approval was required for this study.

## 3. Results

### 3.1. Exploratory Data Analysis

As of 27 April 2020, a total of 629 cases of COVID-19 were diagnosed in Seoul, including two deaths since the first case was confirmed in Seoul on 24 January 2020. More than 60% of all cases were reported in the Seoul metropolitan region, where the proportion of screening is only 1.1% of 9.8 million citizens [22]. We extracted a cluster of more than 20 confirmed cases among all infections caused by community outbreaks from January to December 2020. For example, the cluster size of the Itaewon nightclubs was 139 confirmed cases over 33 days, beginning on 8 May 2020. The cluster of Sarang Jeil church reported the most confirmed cases (641 in 39 days). On average, 16–17 cases were reported each day.

- **Religious Groups**

The largest category, composed of 1178 confirmed cases in Seoul, was linked to religious groups, such as churches and temples. Since the mass infection of the Shincheonji religious organization in Daegu on 18 February 2020, there have been continued infections related to religious facilities including churches, bible meetings, and temples. There was a total of 11 clusters from 25 March to 2 December 2020, including clusters at Sarang Jeil church with 641 cases reported in Seongbuk-gu, Seoul.

- **Convalescent Homes**

The first case of this category in Seoul was identified on 10 June 2020, and five clusters formed by 22 November 2020. This category included clusters that formed in convalescent facilities, such as Hope Daycare Center in Dongdaemun-gu. This category was composed of 215 confirmed cases. The number of confirmed cases related to convalescent homes has surged since June, reaching 102 cases in November 2020.

- **Hospitals**

This category, which comprised 222 confirmed cases in Seoul, was linked to hospitals. As the number of confirmed patients increased due to the second wave, infections in hospitals also increased rapidly since September 2020 and confirmed cases have been steadily occurring as of December 2020. A total of six hospital-related clusters, including the Dobong-gu Dana Hospital-related cluster, occurred from 31 August to 4 December 2020.

- **Workplaces and Schools**

This category was composed of 652 confirmed cases in Seoul. Although there was variation by month, confirmed cases steadily occurred from March to December 2020. There was a total of 15 clusters including a door-to-door sales company cluster, a Richway-related cluster and a Guro-gu call center-related cluster. The transmission related to schools and private academies consisted of four clusters: an acting academy in Gangnam-gu, a seminary in Dongjak-gu, a cram academy related to physical education in Seongbuk-gu and an academy for the examination for teaching licenses in Dongjak-gu.

- **Leisure Facilities**

As of 4 December 2020, in Seoul, 682 confirmed cases had been linked to leisure facilities such as athletic facilities and saunas. The number of confirmed cases has increased since August when the second wave occurred, resulting in a total of 417 in November 2020. There was a total of 17 clusters, including a Gangseo-gu dance lessons-related cluster and a Seocho-gu sauna-related cluster, from 4 March to 4 December 2020.

- **Itaewon Clubs**

From 8 March to 6 June 2020, in Seoul, 139 confirmed cases of COVID-19 were linked to this cluster, which included a person diagnosed with COVID-19 who had visited at least five nightclubs in Itaewon (Yongsan-gu) during the night of May 1 and the dawn of 2 May 2020. According to the KDCA, it was estimated that this patient came into contact with approximately 2000 individuals. Locally transmitted confirmed cases continued to be reported in Seoul, and there were also confirmed cases related to this cluster nationwide, including Gyeonggi and Gangwon regions.

- **Others**

As of 4 December 2020, in Seoul, 168 confirmed cases of COVID-19 were classified as others. This category included clusters related to housing complexes such as Daewoo The O’Ville Plus located in Gangnam-gu and a Guro-gu apartment, as well as a theater company San, Mapo-gu Home Shopping, Yeongdeungpo-gu Nichiren Shoshu Seoul Mission, and Yongsan-gu Armed Forces Welfare Corps.

**Figure 1:**
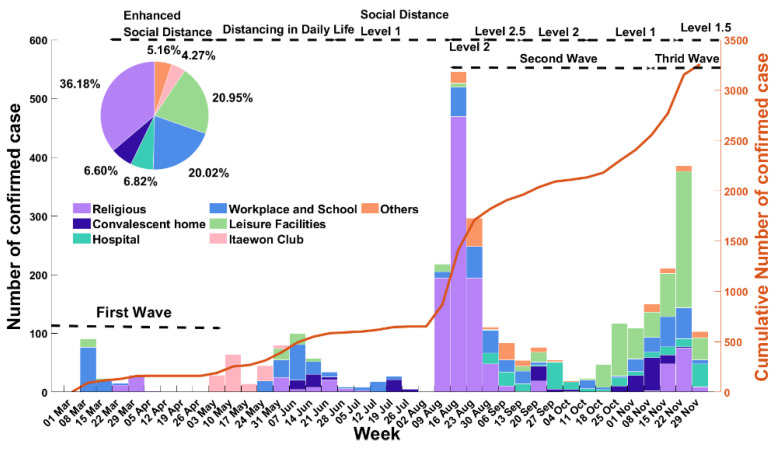
The epidemic curve of weekly confirmed cases of COVID-19 in Seoul, South Korea over the confirmed dates. The pie graph shows the ratio of clusters by category. The red curve represents the cumulative number of confirmed cases corresponding to the left vertical axis.

Figure 1 shows the number of confirmed cases by category and the cumulative number of confirmed cases in Seoul between March and December 2020. The characteristics of the COVID-19 cluster categories are listed in Table 1. The social distancing measures designated by the government were implemented at various levels between level 1 (relaxed social distancing) and level 3 (strict social distancing). Table S1 describes the timeline of the control interventions implemented across the whole country. There have been three waves of epidemic curves in South Korea as of June 2021. The first wave began on February 18 and continued until 5 May 2020, with 637 confirmed cases reported in Seoul and 10,804 nationwide [5,16]. The second wave began on 12 August, followed by the third starting on 13 November. In the second and third waves, the number of confirmed cases in Seoul increased rapidly compared to the first wave, with 6505 and 9716 cases reported, respectively, and 27,942 and 36,332 confirmed cases reported nationwide [5]. Comparing the number of infections every month, Itaewon clubs comprised the largest proportion of cases in May 2020. In addition, the church category that included Sarang Jeil church accounted for the largest proportion in August, and the slope of the cumulative graph also increased sharply in August. These findings indicate that the second wave had already begun in August. Clusters originating from leisure facilities comprised the largest proportion of cases in November, which coincided with the start of the third wave (Figure 1).

### 3.2. Linear Relation between Size and Duration by Clusters

**Figure 2:**
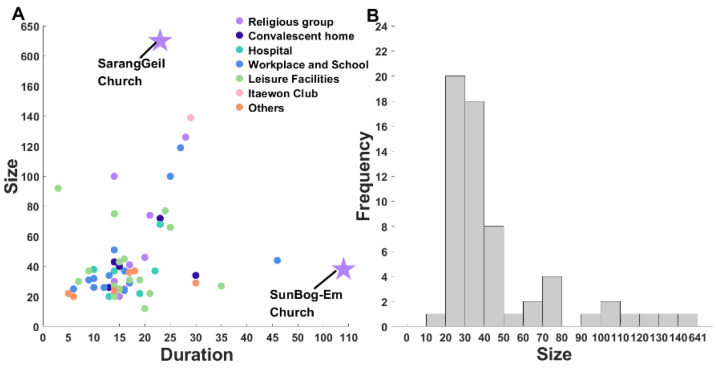
(**A**) The size and duration of 61 clusters categorized by coloring. Sarang Jeil church and Full Gospel church are outliers marked by stars. (**B**) The size and frequency of clusters.

We assumed that duration was the difference of the dates of the first and the last confirmed cases for each cluster. Figure 2A shows the relation between size and duration for 61 clusters. We observed outliers, namely Sarang Jeil church and Full Gospel church, that were large in size (641) and long in duration (109 days). Excluding the outliers, Pearson’s correlation coefficient between size and duration is 0.3442 [23], which indicates that the cluster size is likely larger if the cluster duration is longer. Most durations are concentrated within a timeframe of two weeks. Moreover, Figure 2B describes the corresponding distribution of the frequency for cluster sizes. It was observed that cluster sizes between 20 and 30 accounted for approximately 37.29% of all clusters. A minority of clusters were over 90 in size (10.17%).

### 3.3. The Average Daily Secondary Cases by Cluster

**Figure 3:**
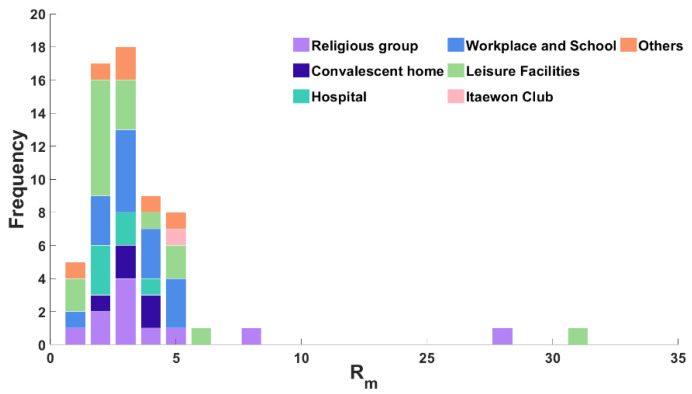
Frequency of mean secondary daily cases for 61 clusters colored by category.

Figure 3 shows the frequency of $R_{m}$, which indicates the average daily secondary cases by cluster. The $R_{m}$ between 2 and 3 was 57.4% for 61 clusters. Additionally, the proportion of frequency of $R_{m}$ for the leisure facility cluster was 1–2, which was lower than other clusters. Clusters with higher $R_{m}$ rarely occurred, except for two cases whose $R_{m}$ were over 25, namely Sarang Jeil church ($R_{m}=26.7083$) and a dance teaching center in the leisure facilities category ($R_{m}=30.6667$). These results show that new daily infections on average were between 2 and 3 for each cluster (Table S2).

**Figure 4:**
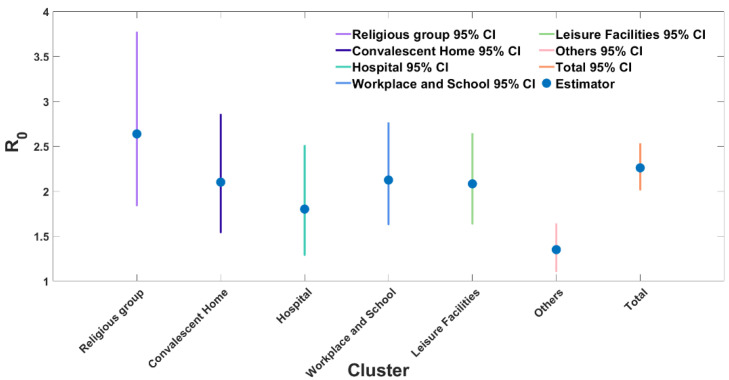
The effective reproduction number with 95% CI estimated by adjusting for the initial cases x=20 from 4 March to 4 December 2020.

When the number of initial cases for each cluster category was 1, $R_{\mathrm{eff}}$ had a value of 20 or more. In particular, the religious cluster has a value of 52.76, which means that on average one primary infection in a cluster causes 52.76 secondary infections. The dispersion parameter κ has a large value greater than or equal to 3, and we estimate that SSEs would not have occurred when the number of initial cases was 1. However, it is unreasonable to assume that the initial case number is 1 as the size of clusters had large values exceeding 100. In particular, the size of the religious category was 1178. It was assumed that the current situation was well reflected when the number of initial cases is 20. When the initial case number was 20, the $R_{\mathrm{eff}}$ had a large value of 2 or more in all categories except hospitals and others. Moreover, the religious cluster including churches had the largest value at 2.64 (95% CI: 1.84–3.78). The $R_{\mathrm{eff}}$ and κ values of all 61 clusters were 2.26 (95% CI: 2.02–2.53) and 0.20 (95% CI: 0.14–0.28), respectively, as shown in Figure 4 and Table 2.

Table 2 compares the estimates of the effective reproduction number and dispersion parameters by cluster, showing that the contagion in clusters is higher than in non-clusters. The values of κ are less than 0.5 in all categories except for the others category. The values are estimated at 0.20 (95% CI: 0.14–0.28) for the total of all clusters and 0.16 (95% CI: 0.06–0.38) for the religious cluster, which are very small values (See Table S3). This result shows that overdispersion has occurred in the cluster. The reproduction number $R_{\mathrm{eff}}$ for the total of all clusters was estimated at 2.26 (95% CI: 2.02–2.53), above the epidemic threshold of 1.0.

Moreover, the previous SARS/MERS outbreaks and current COVID-19 outbreak in other countries were compared in terms of reproduction numbers and dispersion parameters (Refer to Table 3).

Each country’s clusters were observed, including 16 cases originating from a Buddhist worship hall in Hong Kong, 28 cases in Japan linked to a couple returning from Hawaii, and 31 cases in Singapore related to a church gathering. By fitting negative binomial models to empirical cluster size distributions, the maximum likelihood estimators (MLEs) of $R_{0}$ and κ were determined. The estimation values of $R_{0}$ and κ are as follows: 0.61 (90% CI: 0.47–0.78) and 2.30 (90% CI: 0.39–∞) in Hong Kong; 0.48 (90% CI: 0.39–0.59) and 0.51 (90% CI: 0.26–1.42) in Japan; and 0.70 (90% CI: 0.55–0.89) and 1.78 (90% CI: 0.36–∞) in Singapore. The findings suggest that SARS-CoV-2 transmission in Hong Kong, Japan, and Singapore was not overdispersed as of 3 March 2020 (with relatively large values of κ), and thus, there was no strong evidence for the presence of SSEs [13]. COVID-19, which began in January to March in Singapore, was classified into six regional clusters and the reporting delay and reproduction number were predicted. The reproduction number was predicted to be 0.61 (95% CI) by fitting to the negative binomial distribution, and the κ (dispersion number) was predicted to be 0.11.

Our estimate of $R_{\mathrm{eff}}$ for South Korean clusters is much higher than those of Hong Kong (0.61), Japan (0.48), and Singapore (0.70), as of 3 March 2020. For dispersion parameter κ, our result is lower than the estimates (0.51–2.30) of the previous study where there was no strong evidence for the presence of SSEs. The values of the reproduction number and dispersion parameter in Hong Kong, estimated by more recent studies, were 0.74 and 0.33, respectively, indicating that the value of κ was reduced compared to the above study.

Comparing this value with COVID-19 in Hubei, China, and MERS in South Korea, it can be concluded that SSEs have not yet occurred in Singapore; however, the need for public health measures is emerging [15]. COVID-19 cases were reported in Hong Kong from January to April 2020 and were classified into clusters by infection locations; the reproduction number and dispersion number were predicted and the values of 0.58 and 0.43 were derived, respectively. These numbers highlighted that Hong Kong’s constrained community transmission prevented SSEs, while the dispersion number κ in China and Singapore (0.58 and 0.11, respectively) did not [14]. For MERS, where a cluster with a size of over 150 occurred in Korea, the estimates of the reproduction number were 0.47, which is lower than our estimate of $R_{\mathrm{eff}}$. The estimates of the reproduction number in Singapore (0.13) and Beijing (0.94) for SARS were also lower than our result. However, the estimate of κ (0.26) for MERS is greater than our results. In the case of SARS, the values of κ, estimated in Singapore and Beijing, were 0.16 and 0.17, respectively, which are lower than our results.

### 3.4. Monthly Cases by Cluster as of October 2021

**Figure 5:**
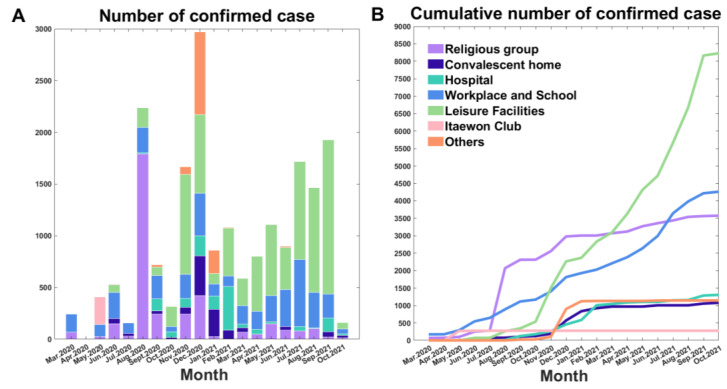
Epidemic curve in South Korea as of October 2021. (**A**). Epidemic curve of monthly cases of confirmed COVID-19 infections in South Korea by confirmed date and colored by cluster category. (**B**). Cumulative curve of monthly cases of confirmed COVID-19 infections in South Korea colored by cluster category.

The transmission dynamics may be influenced by vaccination, which began in South Korea on26 February 2021 [24]. Thus, we focused on the transmission dynamics until December 2020. However, it is important to analyze and compare transmission dynamics for the current cluster-induced transmissions between the years 2020 and 2021. Figure 5 shows the epidemic curve of confirmed cases of COVID-19 by clusters in Seoul, South Korea. In August 2020, approximately 2000 cases caused by the religious group was the largest cluster. In 2021, the number of cases continued to rise between 400 and 1000 from the leisure facilities cluster, while the number of cases caused by the religious groups cluster reduced. The monthly cases by cluster from March 2020 to October 2021 is summarized in Table S4. It can be clearly observed that the characteristic of cluster-caused transmission has changed between 2020 and 2021. Further investigations of cluster-induced transmissions should be carried out. 

## 4. Discussion

We categorized 61 clusters, each of which contained more than 20 cases from 4 March to 4 December 2020 in Seoul, South Korea. We observed transmissions of clusters within religious groups (1178/3256 (36.2%)) most frequently, followed by within leisure facilities (682/3256 (20.9%)) and workplaces and schools (652/3256 (20.0%)). Comparison of the characteristics of clusters in terms of cluster size and infection period revealed that the average infection period in the cluster was approximately two weeks, with Full Gospel church reporting a longer infection period (109 days). Moreover, the Itaewon club cluster had at least 139 cases in a short period of 29 days as the second wave of COVID-19 began in South Korea. The size of the majority of clusters in churches, workplaces and schools ranged between 20 and 40. It can be interpreted that clusters may have formed in large numbers and over longer periods of time, but they were effectively managed owing to better control measures, resulting in a reduced number of cases [17]. Although the government implemented nonpharmaceutical interventions including social distancing in public facilities, transmission is still steadily occurring in the above cluster categories.

The $R_{\mathrm{eff}}$ and κ values of all 61 clusters are 2.26 (95% CI: 2.02–2.53) and 0.20 (95% CI: 0.14–0.28), respectively, as shown in Table 2. The study findings suggest that the reproduction number is larger than 1 in clusters and the distribution of the number of secondary transmissions of COVID-19 revealed overdispersal as κ<1 except for the other clusters. These findings are consistent with those of earlier studies on superspreading events observed during the COVID-19 pandemic [11,13,14,21]. In particular, the religious clusters had the largest value of $R_{\mathrm{eff}}$ among all clusters. Workplaces and schools followed by convalescent home clusters showed high values of $R_{\mathrm{eff}}$. The dispersion parameter κ was also the lowest in the religious cluster (0.16, 95% CI: 0.06–0.38), followed by workplaces and schools (0.20, 95% CI 0.10–0.42) and leisure facilities (0.23, 95% CI: 0.11–0.49).

The reproduction number and dispersion parameter of the current COVID-19 outbreak were compared to those of the previous SARS/MERS outbreaks in Table 3. Overall, their reproduction number was much lower than the present study’s findings of COVID-19, while the dispersion parameter κ was estimated to be a similar value, except for COVID-19 in Singapore ($R_{\mathrm{eff}}=1.78$). In the case of the MERS outbreak, the reproduction number of MERS was much smaller than that of COVID-19, although SSEs did occur.

There are some limitations to this study. First, transmission chains between infectors and infectees in clusters were not clearly observed. Although the previous study during the early stage of the COVID-19 outbreak identified infector–infectee transmission chains, the spread of transmission has made it difficult to find transmission chains [14] as the situation progresses. The superspreading potential of clusters defined using contact tracing data could be underestimated owing to the uncertainty and underreporting of contact tracing for infected cases [14,20]. Therefore, to overcome the unknown information for initial cases by cluster, we estimated the $R_{\mathrm{eff}}$ and κ by varying the number of initial cases by cluster, as shown in Table S3.

Second, since the data are based on the patients’ residential addresses, there may be misclassified cases. Confirmed cases of COVID-19, particularly those individuals who were infected but reside outside Seoul, may have been excluded from the cluster. Further analysis can be conducted if more information on the transmission routes is provided [14].

Third, previous studies estimated the effective reproduction number in real time by incorporating the reporting delays [15] between the illness onset and the diagnosis or reporting using the data of COVID-19 cases for which the dates of symptom onset were available. However, the reporting delays for the confirmed cases were not considered in this study. The purpose of this study was to analyze transmission potential for superspreading events by clusters. Thus, $R_{\mathrm{eff}}$ and κ were jointly estimated, including COVID-19 cases for which symptom onset dates were not available to determine the clusters accurately.

Although the offspring distributions of cluster size are useful, they are not directly applicable to an ongoing outbreak as the final cluster size has yet to be determined. If superspreading events were missed during the outbreak, then these estimates could be inaccurate. However, in the present study, the clusters could successfully be classified as the public data provided the source of infection of confirmed cases, such as churches, schools and workplaces. This is the first attempt to identify the characteristics of clusters in Seoul, South Korea, by estimating the reproduction number and dispersion parameters. This may also be used to estimate cluster sizes, and to conduct further research on cluster-specific basic reproduction number for COVID-19 outbreaks [11,13,14,15]. The study supports the possibility of highly overdispersed transmission of COVID-19. Therefore, since the transmission of SARS-CoV-2 is still ongoing, the transmission potential for clusters should be continuously analyzed to control massive infection effectively in future COVID-19 scenarios.

## 5. Conclusions

We have investigated the characteristics of cluster-induced transmissions in Seoul, South Korea from March to December 2020. Since the majority of the clusters in South Korea are focused in Seoul, it is essential to elucidate the characteristics of cluster-induced transmissions in Seoul. A total of 61 clusters has been identified, and furthermore, we have analyzed 61 clusters by estimating the cluster-specific basic reproduction number and dispersion parameter. Our results showed that the cluster-specific basic reproduction number was high and the dispersion parameter was small enough (highly overdispersed). These findings confirmed that superspreading events were one of the common features of the Seoul COVID-19 outbreaks. In addition, the overall effective reproduction number for the clusters is estimated at 2.26, which is higher than previous SARS-CoV and MERS-CoV outbreaks. For clusters originating from religious groups such as churches and temples, our estimates of the reproduction number and dispersion parameter are 2.64 and 0.16, respectively. It is clearly observed that the strength of SSEs was higher in the religious groups. Since most cases do not contribute to the expansion of transmission, effective reproduction numbers can be rapidly reduced by preventing SSEs in such groups. Therefore, these measurements can provide insights into the potential risk of superspreading events of ongoing COVID-19 outbreaks and effective interventions to prevent massive cluster-induced transmissions.

## Figures and Tables

**Table 1:** Characteristics of COVID-19 cluster categories in Seoul, South Korea as of December 4, 2020 excluding “Others” category.

Categories	Infection Locations	Cluster Size	Confirmed Date for the First Case Linked to Cluster	Confirmed Date for the Last Case Linked to Cluster
Religious Groups	Church, bible meeting, temple	1178	25 March 2020	2 December 2020
Convalescent Homes	convalescent facility	215	10 June 2020	22 November 2020
Hospitals	private hospital, university hospital	222	31 August 2020	4 December 2020
Workplaces and Schools	Call center, office, city office, school, academy, distribution center	652	8 March 2020	4 December 2020
Leisure Facilities	Athletic facility, Korean sauna, private meeting, market, teaching center, internet cafe	682	4 March 2020	4 December 2020
Itaewon Clubs	Nightclub	139	8 May 2020	6 June 2020

**Table 2:** Inference results for comparing the effective reproduction number and dispersion of clusters as initial case = 20 for COVID-19 in South Korea.

	Religious Group	Convalescent Home	Hospital	Workplace and School	Leisure Facilities	Others	Total
$R_{\mathrm{eff}}$	2.64 (1.84–3.78)	2.10 (1.54–2.86)	1.80 (1.29–2.51)	2.12 (1.63–2.76)	2.08 (1.64–2.65)	1.35 (1.11–1.64)	2.26 (2.02–2.53)
κ	0.16 (0.06–0.38)	0.50 (0.11–2.22)	0.34 (0.09–1.27)	0.20 (0.10–0.42)	0.23 (0.11–0.49)	2.46 (0.13–33.73)	0.20 (0.14–0.28)

The numbers in parentheses represent the 95% profile likelihood confidence intervals.

**Table 3:** Inference results for comparing the effective reproduction number and dispersion of COVID-19 in other countries or other epidemics.

Virus	Epidemics	$R_{eff}$	κ	References
SARS-CoV2	Republic of Korea 2020	2.26 (95% CI: 2.02–2.53)	0.20 (95% CI: 0.14–0.28)	Estimated
	Hong Kong 2020	0.61 (90% CI: 0.47–0.78)	2.30 (90% CI: 0.39–∞)	[13]
	Japan 2020	0.48 (90% CI: 0.39–0.59)	0.51 (90% CI: 0.26–1.42)	[13]
	Singapore 2020	0.70 (90% CI: 0.55–0.89)	1.78 (90% CI: 0.36–∞)	[13]
	Hong Kong 2020	0.74 (95% CI: 0.58–0.97)	0.33 (95% CI: 0.14–0.98)	[14]
MERS-CoV	Republic of Korea 2013	0.47 (95% CI: 0.29–0.80)	0.26 (90% CI: 0.11, 0.87)	[20]
SARS-CoV	Singapore 2003	0.13 (90% CI: 0.54–2.65)	0.16 (90% CI: 0.11–0.64)	[18]
	Beijing 2003	0.94 (90% CI:0.10–0.64)	0.17 (0.10–0.64)	[18]

## Data Availability

The COVID-19 epidemiological data in Seoul province is available in the report formulated by the Seoul Metropolitan Government.

## Supplementary Materials

The following are available online at https://www.mdpi.com/article/10.3390/ijerph182412893/s1, Figure S1. Epidemic curve in South Korea. Table S1. Administrative measures in South Korea from March to December 2020. Table S2. The frequency of mean secondary daily cases ($R_{m}$) by clusters. Table S3. Inference results for the effective reproduction number and the dispersion parameter of clusters for various numbers of initial cases for COVID-19 in Seoul, South Korea. Table S4. Describes the number of COVID-19 clusters with the case numbers range by month and year from March 2020 to October 2021 in Seoul metropolitan area.
