# Supplementary material for: Analysis of Superspreading Potential from Transmission Clusters of COVID-19 in South Korea
Source: Int J Environ Res Public Health. 2021 Dec 7;18(24):12893. doi: 10.3390/ijerph182412893 (PMC8701974; doi:10.3390/ijerph182412893)
Supplement: Supplementary file 1 [file ijerph-18-12893-s001.zip › ijerph-1423856-supplementary.pdf]

International Journal of
Environmental Research and Public Health

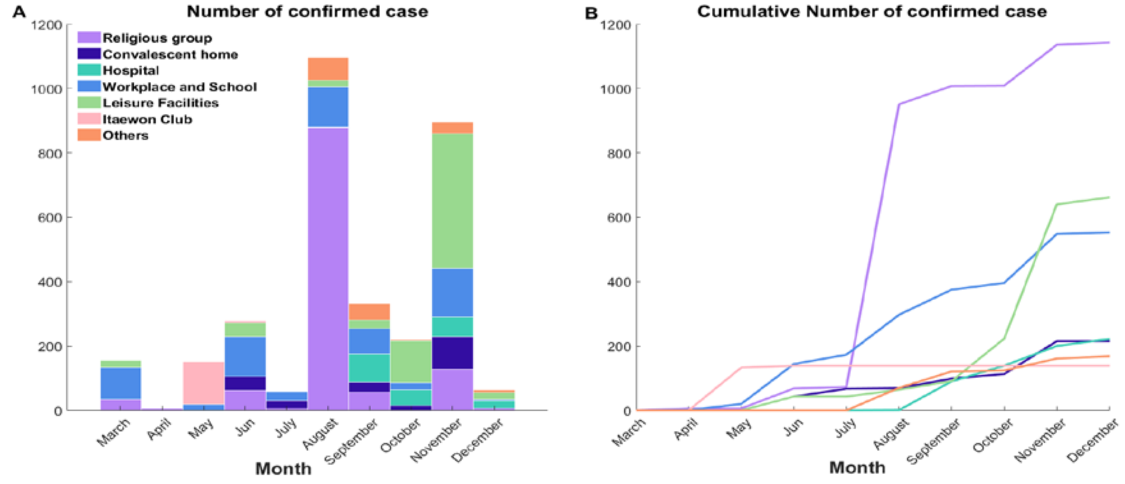

Figure S1. Epidemic curve in South Korea. A. Epidemic curve of monthly cases of confirmed COVID-19 infections in South Korea by confirmed date and colored by cluster category. **B.** Cumulative curve of monthly cases of confirmed COVID-19 infections in South Korea colored by cluster category.

Table S1. Administrative measures in Korea from March to December, 2020

Date	Administrative measures	Reference
March 22	Enhanced social distancing	KDCA COVID-19 Response [26]
April 1	Self-quarantine for arrivals from all overseas countries for 2 weeks	KDCA COVID-19 Response [26]
June 28	Introduction of three-level social distancing<br>Enforced social distancing level 1	KDCA COVID-19 Response [26]
August 16	Enforced social distancing level 2 (Seoul, Gyeonggi)	KDCA COVID-19 News & Issues [27]
August 21	Prohibition of rallies of 10 or more people in Seoul	KDCA COVID-19 News & Issues [27]
August 21	Allow church services only as non-face-to-face in metropolitan area including Seoul, Gyeonggi, and Incheon	KDCA COVID-19 News & Issues [27]
August 23	Enforced social distancing level 2	KDCA COVID-19 Response [26]
August 30	Enhanced social distancing to level 2.5 in metropolitan area	KDCA COVID-19 Response [26]
September 14	Relaxed social distancing to level 2 in metropolitan area	KDCA COVID-19 Response [26]

September 25	Maintain prohibition of gathering for high-risk facilities (11 types) in metropolitan area	KDCA COVID-19 News & Issues [27]
October 12	Relaxed social distancing to level 1<br>Maintain social distancing level 2 in some metropolitan area	KDCA COVID-19 News & Issues [27]
November 07	Relaxed social distancing to level 1 nationally	KDCA COVID-19 News & Issues [27]
November 19	Enforced social distancing level 1.5 in metropolitan area and some areas of Gangwon-do	KDCA COVID-19 News & Issues [27]
November 23	Enforced social distancing level 2 in metropolitan area	KDCA COVID-19 News & Issues [27]

In social distancing level 2, gatherings of 50 people indoors or more than 100 outdoors are prohibited, and gatherings of 10 or more people in level 3 are prohibited.

Table S2. Comparison of frequency of mean secondary daily cases (R_m) by clusters.

R_m	Religious group	Convalescent home	Hospital	Workplace and School	Leisure Facilities	Itaewon Club	Others	Total
0-1	1 (9.09%)	-	-	1 (6.67%)	2 (11.76%)	-	1 (16.67%)	5
1-2	2 (18.18%)	1 (20%)	3 (50%)	3 (20%)	7 (41.18%)	-	1 (16.67%)	17
2-3	4 (36.36%)	2 (40%)	2 (33.33%)	5 (33.33%)	3 (17.65%)	-	2 (33.33%)	18
3-4	1 (9.09%)	2 (40%)	1 (16.67%)	3 (20%)	1 (5.88%)	-	1 (16.67%)	9
4-5	1 (9.09%)	-	-	3 (20%)	2 (11.76%)	1 (100%)	1 (16.67%)	8
5-6	-	-	-	-	1 (5.88%)	-	-	1
6-7	-	-	-	-	-	-	-	-
7-8	1 (9.09%)	-	-	-	-	-	-	1
28-29	1 (9.09%)	-	-	-	-	-		1
29-30	-	-	-	-	-	-	-	-
30-31	-	-	-	-	-	-	-	-
31-32	-	-	-	-	1 (5.88%)	-	-	1
Total	11(18.03%)	5(8.20%)	6(9.84%)	15(24.59%)	17(27.87%)	1(1.64%)	6(9.84%)	61

Table S3. Inference results for comparing the effective reproduction number and dispersion of clusters as initial cases (x = 1,3,5,15,20) for COVID-19 in South Korea.

	Initial (index)	R_{eff}	κ
Religious group	1	52.76 (36.74-75.23)	3.15 (1.29-7.51)
	3	17.59 (12.27-25.14)	1.05 (0.43-2.53)
	5	10.55 (7.36-15.09)	0.63 (0.26-1.52)
	15	3.51 (2.46-5.03)	0.21 (0.09-0.51)
	20	2.64 (1.84-3.78)	0.16 (0.06-0.38)
Convalescent home	1	42.03 (30.81-57.08)	10.02 (2.04-37.35)
	3	14.01 (10.28-19.06)	3.32 (0.72-14.06)
	5	8.40 (6.17-11.44)	1.99 (0.44-8.65)
	15	2.80 (2.06-3.81)	0.66 (0.15-2.96)
	20	2.10 (1.54-2.86)	0.50 (0.11-2.22)
Hospital	1	36.03 (25.77-50.16)	6.85 (1.75-23.34)
	3	12.01 (8.60-16.75)	2.28 (0.60-8.27)
	5	7.20 (5.16-10.05)	1.37 (0.36-5.03)
	15	2.40 (1.72-3.35)	0.46 (0.12-1.70)
	20	1.80 (1.29-2.51)	0.34 (0.09-1.27)
Workplace and School	1	42.49 (32.60-55.20)	4.03 (1.89-8.38)
	3	14.16 (10.88-18.42)	1.34 (0.63-2.82)
	5	8.50 (6.53-11.06)	0.81 (0.38-1.70)
	15	2.83 (2.18-3.69)	0.27 (0.13-0.57)
	20	2.12 (1.63-2.76)	0.20 (0.10-0.42)
Leisure Facilities	1	41.64 (32.73-52.84)	4.64 (2.18-9.62)
	3	13.88 (10.92-17.63)	1.55 (0.73-3.24)
	5	8.32 (6.55-10.58)	0.93 (0.44-1.95)
	15	2.78 (2.18-3.53)	0.31 (0.15-0.65)
	20	2.08 (1.64-2.65)	0.23 (0.11-0.49)
Others	1	27.00 (22.19-32.81)	54.25 (1.13-99.20)
	3	9.00 (7.40-10.94)	16.85 (0.68-85.73)
	5	5.40 (4.44-6.57)	9.98 (0.45-73.08)
	15	1.80 (1.48-2.19)	3.29 (0.16-41.16)
	20	1.35 (1.11-1.64)	2.46 (0.13-33.73)
Total	1	45.20 (40.34-50.63)	4.00 (2.89-5.50)
	3	15.07 (13.45-16.88)	1.33 (0.97-1.84)
	5	9.04 (8.07-10.13)	0.80 (0.58-1.10)
	15	3.01 (2.69-3.38)	0.27 (0.19-0.37)
	20	2.26 (2.02-2.53)	0.20 (0.14-0.28)
